# Dynamic Sensor-Based Data Management Optimization Strategy of Edge Artificial Intelligence Model for Intelligent Transportation System

**DOI:** 10.3390/s25072089

**Published:** 2025-03-26

**Authors:** Nu Wen, Ying Zhou, Yang Wang, Ye Zheng, Yong Fan, Yang Liu, Yankun Wang, Minmin Li

**Affiliations:** 1 Internet of Things Research Institute, Shenzhen Polytechnic University, Shenzhen 518055, China; wennu1989@szpu.edu.cn (N.W.); wangyang@szpu.edu.cn (Y.W.); fanyong@szpt.edu.cn (Y.F.); 2 Key Laboratory of Urban Land Resources Monitoring and Simulation, Ministry of Natural Resources, Shenzhen 518055, China; 3 School of Information Science and Engineering, Shandong Agricultural University, Tai’an 271018, China; zhouying@sdau.edu.cn; 4 Faculty of Electrical Engineering and Computer Science, Ningbo University, Ningbo 315211, China; zhengye@nbu.edu.cn; 5 Hefei Institutes of Physical Science, Chinese Academy of Sciences, Hefei 230031, China; liuy@aiofm.ac.cn; 6 Guangdong Laboratory of Artificial Intelligence and Digital Economy (SZ), Shenzhen 518123, China

**Keywords:** intelligent transportation, artificial intelligence, sensor-based data

## Abstract

In the intelligent transportation field, object recognition, detection, and location applications face significant real-time challenges. To address these issues, we propose an automatic sensor-based data loading and unloading optimization strategy for algorithm models. This strategy is designed for artificial intelligence (AI) application systems that leverage edge computing. It aims to solve resource allocation optimization and improve operational efficiency in edge computing environments. By doing so, it meets the real-time computing requirements of intelligent transportation business applications. By adopting node and sensor management mechanisms as well as efficient communication protocols, dynamic sensor-based data management of AI algorithm models was achieved, such as pedestrian object recognition, vehicle object detection, and ship object positioning. Experimental results show that while maintaining the same recall rate, the inference time is reduced to one tenth or even one twentieth of the original time. And this strategy can enhance privacy protection of sensor-based data. In the future research, we may consider integrating distributed computing under high load conditions to further optimize the response time of model loading and unloading for multi-service interaction, and enhance the balance and scalability of the system.

## 1. Introduction

With the rapid development of edge computing technology, more and more intelligent transportation application scenarios require the deployment of artificial intelligence algorithm models on edge devices to meet real-time response, reduce network bandwidth usage, and enhance data privacy protection [1,2,3,4]. However, edge devices often face limited computing resources and storage space, making it challenging to support the parallel loading and execution of numerous models [5,6,7,8]. To address these challenges, this study proposes an efficient automatic loading and unloading strategy for artificial intelligence models, which is designed to optimize the resource utilization and operational efficiency of edge devices, thus enhancing the overall performance of the intelligent transportation system.

Joint optimization of task offloading and resource allocation has emerged as a research hotspot in edge computing [9,10,11,12]. Existing studies on this topic typically assume tasks are offloaded to a single edge server, with each edge device generating one task by default. Even when multiple servers are considered, studies often overlook load balancing among them [1]. At least four aspects are included for video or image processing of sensor-based data in the process of edge-based artificial intelligence application in intelligent transportation systems: edge node and sensor management, cloud-edge collaborative communication, video management and streaming, and artificial intelligence model application.

In the field of edge node and sensor management, Zhang Peng et al. [2] distributed the multi-agent joint action policy function across individual agent devices using a multi-agent reinforcement learning value decomposition method, thereby facilitating joint offloading decisions for the system. This allows the system to adaptively modify its strategy for edge upload or local computation during joint computation offloading tasks, considering the current computing and communication capabilities of the agent devices, the task’s delay requirements, communication data volume, and computational demands. Additionally, researchers have developed other edge caching strategies to enhance node optimization, including dynamic virtual edge node caching schemes that generate edge node caching strategies based on predictive information, and service caching cost strategies for single-node limited-service time intervals [3,4].

As for cloud-edge collaborative communication mechanism of sensor-based data, Wu Xuewen et al. [5] introduced a game theory-driven approach for resource allocation and task offloading within a multi-user system that incorporates cloud-edge collaboration. This approach optimizes the allocation of computing resources, uplink power, and task offloading decisions to minimize latency, energy consumption, and computational costs. Alonso et al. [6] argued that it is the surge in mobile terminal devices and computing tasks, combined with limited network bandwidth and extended transmission distances that significantly impedes the data transmission of computing tasks. This leads to increased energy consumption and latency during transmission, ultimately failing to meet the requirements of delay-sensitive computing tasks and compromising the quality of user experience [6]. Deng Wengui et al. [7] proposed an edge cloud collaborative task offloading strategy based on deep reinforcement learning. By establishing a network model, communication model, and computing model under the edge cloud collaborative architecture, the system aims to minimize latency and energy consumption, and design a DQN offloading strategy based on deep reinforcement learning.

In the field of video management and streaming, Li et al. [8] introduced a mobile edge computing video caching strategy that employs branching and binding algorithms to optimize issues, effectively lowering system latency and energy consumption. Tan et al. [9] examined the issues of offloading decisions, collaborative decisions, and the allocation of computing and communication resources within multi-user cooperative mobile edge computing networks, addressing the task allocation challenges of scheduling edge computing resources for multiple users. To address the issues of data drift, model evolution speed, and average inference accuracy in online evolution, and to enhance the precision and speed of model evolution in multi-terminal collaboration, Wang Lehao et al. [10] proposed a method and system for the co-evolution of intelligent recognition models for multi-terminal video streams based on the concept of integrating software and hardware.

In the context of artificial intelligence in transportation application systems, Chowdhury et al. [11] introduced a user preference-aware computing task offloading and bandwidth resource allocation scheme by considering the varying sensitivities of different business tasks to latency, which effectively reduced task execution time and conserving system costs. Aiming to minimize service caching and maximize user satisfaction, Li Wei et al. [12] employed constraints such as drone and user energy consumption, along with information causality, to jointly optimize service caching, user offloading strategies, time partitioning, computing resource allocation, and UAV flight trajectories, framing it as a nonlinear mixed-integer programming problem. Additionally, a prevalent method involves employing pruning networks [13,14,15,16] for processing when implementing artificial intelligence algorithms at the edge. For example, the Edge Popup [13] algorithm was proposed to find sub models with high accuracy during initialization, and the LFPC [14] algorithm was proposed to use predefined pruning strategies to adaptively select appropriate pruning standards for different functional layers.

To facilitate automated loading and unloading of sensor-based data with AI algorithm models, the system integrates advanced strategy implementation mechanisms. This paper employs methods such as node and sensor management mechanisms, real-time video streaming transmission strategies, cloud-edge collaborative communication mechanism of sensor-based data, and intelligent decision-making configuration for intelligent transportation application. Based on historical data and real-time information, node resources and task requirements are selected to achieve optimal model loading and unloading strategies for sensor-based data. Additionally, the system is equipped with efficient communication protocols and data synchronization mechanisms to ensure real-time and accurate cloud-edge sensor-based data transmission and AI model updates.

This paper focuses on the dynamic sensor-based data loading and unloading of artificial intelligence models in the edge computing environment, aiming to optimize model management in intelligent transportation based on real-time requirements and environmental conditions. It constructs an application model library encompassing various types and functions of artificial intelligence models, and develops a comprehensive suite of edge computing platform application systems and sensor-based data dynamic process strategies for intelligent transportation applications. By monitoring the resources and network status of edge devices, as well as task requirements, it intelligently selects and loads suitable models. This addresses and optimizes the issues of inefficient resource allocation and model compatibility that are commonly encountered by existing methods in practical intelligent transportation applications.

Key contributions of this paper:

(1) Innovative Strategy: The study proposes a novel model automatic loading and unloading strategy based on reinforcement learning (RL), which endows the system with dynamic decision-making capabilities. This strategy enables the system to adaptively adjust resource allocation in real time, ensuring optimal performance under varying workloads and environmental conditions.
(2) Addressing Research Gaps: The research addresses the limitations of prior studies by achieving efficient resource allocation across cloud and edge devices. This approach significantly enhances the performance of various functional components, bridging the gap between theoretical models and practical implementations.
(3) System Construction: The authors construct a comprehensive application model library and develop an edge computing platform system, exemplified by its application in an intelligent transportation system (ITS). Additionally, a dynamic data processing strategy is designed to enable seamless integration and coordination between cloud and edge infrastructures.
(4) Performance Optimization: The proposed framework significantly improves overall resource utilization and reduces task delays. By decoupling real-time processing tasks from information transmission tasks, edge devices can efficiently handle real-time computations while transmitting critical data to the cloud. The automatic resource allocation strategy introduced in this study achieves a balance between resource efficiency and model performance, ensuring robust and scalable system operations.

This paper primarily discusses the dynamic sensor-based data management optimization strategy of edge artificial intelligence model for intelligent transportation systems. The paper consists of the following Sections: Related Work, Edge Artificial Intelligence Applications for Intelligent Transportation Systems, Key Technologies of the System, Application Cases of AI Technology in Intelligent Transportation Systems, Summary, and Future Work.

## 2. Related Work

### 2.1. Background of Edge Computing

In recent years, edge computing has made significant progress and has become a driving force in the transformation of information technology. With the exponential growth of Internet of Things (IoT) devices and the increasing demand for real-time data processing in various applications, the limitations of traditional cloud computing architectures have become increasingly apparent. In the traditional cloud computing model, data must be transmitted to remote cloud servers for processing, which not only results in high network latency but also places significant pressure on network bandwidth. Particularly in fields such as intelligent transportation, industrial automation, and smart healthcare, where real-time performance and data privacy are critical, such delays can lead to severe consequences, such as increased traffic congestion, higher risks of industrial accidents, and delays in medical diagnosis [17].

Edge computing addresses these challenges by moving computational tasks closer to the data source at the edge of the network, significantly reducing data processing paths and minimizing latency. At the same time, edge computing reduces the transmission of data over public networks, thereby enhancing data privacy protection. For example, in intelligent transportation systems, many sensors deployed on roads can collect real-time traffic flow and vehicle speed data. Edge computing devices can process and analyze this data locally, enabling timely adjustments to traffic signal timing and optimizing traffic flow without the need to transmit all data to the cloud [18]. This distributed computing architecture significantly improves the overall performance and reliability of systems, meeting the urgent demand for efficient and real-time data processing in modern digital societies and providing strong support for innovation and development in various application domains [19].

### 2.2. AI Models in Edge Computing

The integration of artificial intelligence (AI) models into edge computing environments further expands their potential and value. AI models, particularly deep learning neural networks, are capable of efficiently analyzing and predicting complex data. Deploying these models on edge devices enables real-time intelligent processing of local data, providing more timely and accurate decision-making support for applications [20]. For instance, in intelligent transportation systems, AI models can analyze real-time traffic flow data, predict congestion, and dynamically adjust traffic signal control strategies to optimize traffic flow [21].

However, deploying AI models on edge devices faces several challenges. First, edge devices typically have limited computational resources, such as low CPU performance, limited memory, and storage capacity, making it difficult to support the operation of large-scale and complex AI models [22]. Second, the loading and updating of models consume significant resources and network bandwidth, making it crucial to achieve efficient model loading, unloading, and dynamic management under resource-constrained conditions [23]. Additionally, the heterogeneity of hardware architectures and operating systems across different edge devices poses challenges in ensuring the compatibility of AI models with various devices [24].

Existing research has made some progress in the application of AI models in edge computing. For example, model compression techniques such as pruning and quantization can reduce model size and computational requirements, enabling them to run on edge devices [22]. Furthermore, lightweight AI models have been specifically designed and optimized for edge computing environments, achieving a balance between model performance and resource consumption [25]. Nevertheless, there are still many challenges in the efficient management and application of AI models in edge computing environments. This study aims to propose innovative solutions to these problems, promoting the deep integration and application of edge computing and AI models in fields such as intelligent transportation.

## 3. Edge Artificial Intelligence Application for Intelligent Transportation System

This paper investigates the application of edge-side artificial intelligence (AI) models in the context of intelligent transportation systems (ITS). Using the specific case of a ship-bridge collision scenario as an example, the study provides a detailed analysis of how AI models can be effectively deployed and executed on low-computing-power edge devices. The research focuses on optimizing the performance of AI algorithms under resource-constrained conditions, ensuring real-time processing and decision-making capabilities. By addressing the challenges of computational limitations and latency, this work contributes to the advancement of edge AI in critical transportation applications, offering practical insights for enhancing safety and efficiency in intelligent transportation systems.

The edge computing platform for intelligent transportation systems encompasses a comprehensive “sensor, edge, cloud, and intelligence” management framework. It extends cloud applications to the edge by connecting to edge nodes, integrating sensor-based data between the edge and cloud, and fulfilling user requirements for remote management, data processing, analysis, decision-making, and intelligent edge computing resource allocation. Additionally, the platform provides unified capabilities for edge node/application monitoring, log collection, and other operational and maintenance functions in the cloud, offering enterprises a holistic edge computing solution that integrates cloud and edge computing services.

For easy of understanding, the relevant special names and explanations in this article are shown in Table 1.

The edge computing platform system emphasizes IoT sensor access and cloud-edge collaborative processing capabilities. It includes interactive functions such as edge node registration and activation, edge node information management, edge application operation management, terminal sensor access management, and terminal sensor information management. The system supports various basic edge computing capabilities, including security verification, sensor access, resource allocation, cloud-edge collaboration, and data processing for diverse applications. By achieving deep decoupling between business development and resource scheduling, the system accelerates business development efficiency while enhancing job processing capabilities and quality

## 4. Key Technologies of the Dynamic Sensor-Based Data Management Optimization Strategy

### 4.1. Node and Sensor Management

A management approach for edge nodes and edge sensors is implemented based on the edge computing backend system and the cloud-edge collaboration system to reduce the communication costs and complexities between those two systems. This primarily includes interactive functions such as edge node registration and activation, edge node information management, edge node operation management, terminal sensor access management, and terminal sensor information management.

The management and operation of nodes and sensors are both operated by users in the browser, facilitating the processing of node and sensor configuration information. Node configuration information includes attribute information (id, serial code, database and sensor related information) and monitoring information (CPU/GPU/memory/bandwidth and other data information), while sensor configuration information includes attribute information (id, sensor identification code, node and application related information) and list information (sensor list and sensor status information).

Assuming there are *m* edge nodes and *k* sensors in the application system, represented by sets *N* and *S* respectively, where $N=\left\{n_{1},n_{2},\;\ldots ,\;n_{m}\right\}$, $S=\left\{s_{1},s_{2},\;\ldots ,\;s_{k}\right\}$. As described above, we consider the GPU/CPU/memory parameters of the device as an efficiency evaluation for loading and unloading.

When node *i* executes the *k*th task, the local latency in the application system can be expressed as:

(1) $$t_{i,k}=\frac{c_{i}^{k}}{f_{i}}$$

Here, $f_{i}$ represents the computing power of node *i* and $c_{i}^{k}$ represents the number of computing cycles required for the current CPU task $t_{i,k}$.

**Figure 1:**
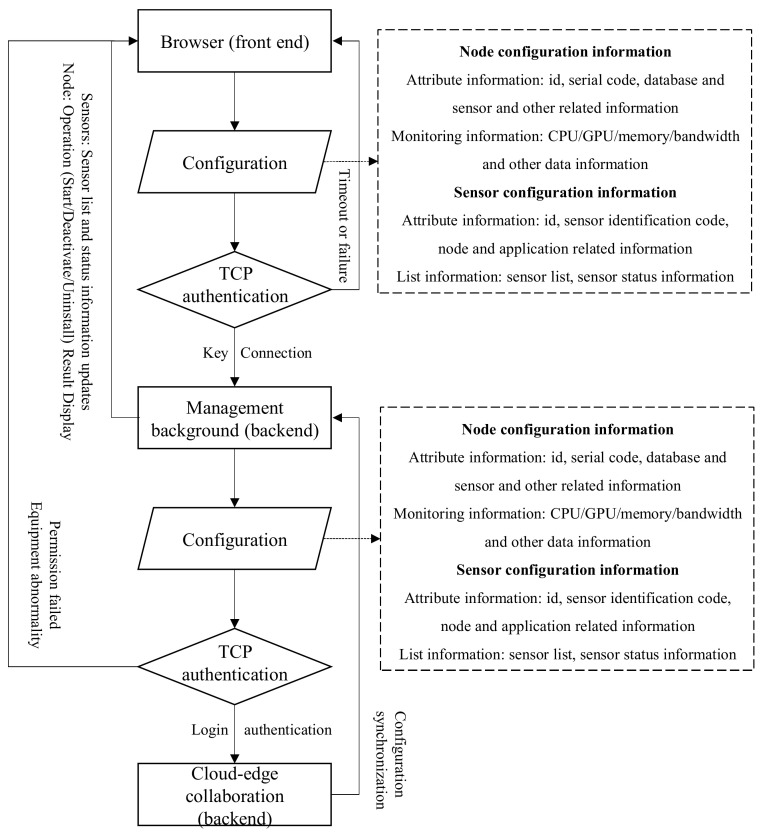
Node and sensor management process.

Our execution rules will follow the process shown in Figure 1, while considering factors such as task latency and resource usage for task loading and unloading. As shown in Figure 1, the communication process and data transmission process between sensors and edge nodes have been proposed, and we provide a detailed introduction to the implementation method of users sending requests and operating nodes or sensors, which specifically includes the following two parts.

#### 4.1.1. Communication Between Browser and Management Background

(1) Node management: The user sends a node operation (start, stop, or uninstall). After the two services successfully establish a connection, the edge computing background system actively requests to obtain all online edge node information of the cloud-edge collaborative system, and matches the edge node information in the database according to the edge node id to determine whether the edge node is in the library. If it is already in the library, the information is synchronized; if the node information does not exist in the library, the node information is encapsulated and then the library operation is performed. At the same time, the association information between the user and the edge node is recorded in the database to realize the binding relationship of users on the Web business side of the edge computing background system. After the edge node is successfully activated, the node is officially enabled and subsequent operations can be performed on the node in the web list.

(2) Sensor management: When users operate terminal sensors on the Web page, the edge computing background system matches the terminal sensor ID and edge node ID in the request parameters with the terminal sensor ID and edge node ID in the binding relationship table in the database. If the match is successful, it means that the binding relationship is correct and the data are legal. Then the user’s operation instructions on the terminal sensor on the Web page can be sent to the cloud-edge collaborative system to realize remote operation of the terminal sensor. The newly added edge terminal sensor needs to be configured with communication protocols, IP, ports and other information to provide users with subsequent sensor node screening and binding functions.

#### 4.1.2. Communication Between Management Backend and Cloud-Edge System

(1) Node management: The edge computing backend system encapsulates the edge node ID and operation instructions sent by the web page and sends them to the cloud-edge collaborative system. First, it authenticates the binding relationship between the user and the edge node based on the binding relationships stored in the database. It then performs preliminary validation checks on the current state of the edge node and the operation instructions. Operations that comply with the edge node’s business logic, such as rebooting and deletion, are sent to the cloud-edge collaborative system.

(2) Sensor management: The edge computing backend system encapsulates data according to the instruction format agreed upon with the cloud-edge collaborative system and sends it to the cloud-edge collaborative system for the formal binding of terminals and nodes. The cloud-edge collaborative system returns the binding results to the edge computing backend, where the backend system records the binding status in the database based on the feedback. The binding results of terminal sensors and edge nodes are ultimately displayed on the web page. When changes to the corresponding sensor are needed, such as attribute changes, sensor activation, or deactivation, the edge computing backend system can implement attribute changes, activation, and unloading operations on the sensor based on the information that needs to be changed and the agreed instructions with the cloud-edge collaborative system.

(3) Information synchronization: The cloud-edge collaborative system continuously sends data such as sensor status and performance indicators to the edge computing backend system. The backend system analyzes and compiles the information on sensor status and performance indicators based on the edge node ID and edge sensor ID in the received data, and displays it on the web page, achieving timely management of edge sensors.

### 4.2. Application Deployment of Artificial Intelligence Models

**Figure 2:**
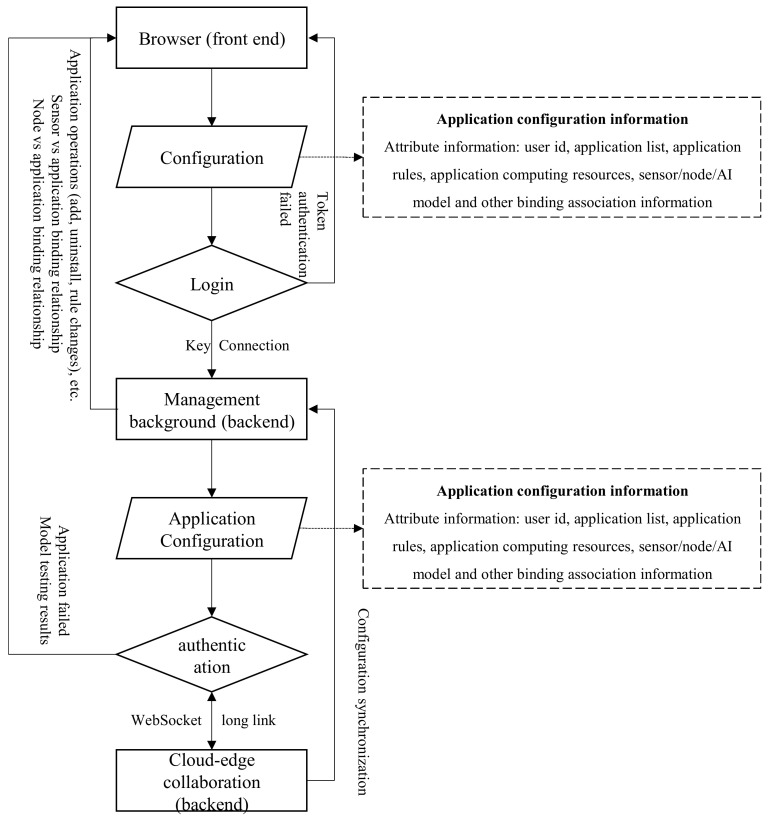
Application of artificial intelligence models in intelligent transportation.

The goal is to reduce the communication cost and complexity between the edge computing backend system and the cloud-edge collaborative system, as well as the selection and maintenance of algorithms. This includes functionalities such as application addition, algorithm management, terminal sensor access, terminal node management, and detection rule settings. As shown in Figure 2, frontend management in a browser runs on edge devices, while backend configuration requires cloud-edge servers to run and manage. And the business application of AI models in the edge computing of intelligent transportation backend system involves the following steps:

User Application Configuration Operations: When using AI models as the core algorithm in edge computing business scenarios, users must select algorithms and models corresponding to their industry to proceed with subsequent operations such as node device binding and activation. Users retrieve all online edge application information from the cloud-edge collaborative system, match it with edge application information in the edge system’s database, and verify whether the edge application information is recorded in the database based on the edge application ID. If the ID does not exist in the database, it is recorded. Otherwise, the latest information is synchronized, such as updating the corresponding online status or configuration information. This achieves data consistency of edge applications on both ends.

Application Rule Changes and Resource Allocation: After successfully activating the edge application, the algorithm application is formally activated and can be operated on the web list. The cloud-edge collaborative system continuously monitors the running status of the edge applications and actively pushes real-time detection results (e.g., personnel trespassing, ship deviation, vehicle status) and abnormal results (e.g., task failure, false/missed detections) as scenario screenshots to the edge computing backend system through the successfully connected WebSocket link. When application configuration rules need to be changed, the edge backend system encapsulates the latest rule configuration data according to the agreed communication protocol with the cloud-edge collaborative system and synchronizes it to the cloud-edge collaborative system. The cloud-edge collaborative system receives the instructions and performs operations such as issuing, starting, stopping, and changing the application on different nodes based on the current node resource usage and application resource configuration, and sends the change and deployment results to the edge computing backend system, displaying them on the web page list of the edge computing backend system.

Application process of artificial intelligence model for video object detection: Users select a camera from the list of terminal cameras provided on the web page of the edge computing backend system to start viewing the video. During this period, users can take screenshots of the footage, and enable or disable the camera. The edge computing backend system encapsulates the operation information according to the agreed instructions and sends it to the cloud-edge collaborative system. The cloud-edge collaborative system issues the corresponding instructions to edworker based on the instructions and camera property configuration. After receiving the instructions and parsing out the camera ID, edworker finds the corresponding camera based on the ID and sends the corresponding operation instructions to the camera.

**Figure 3:**
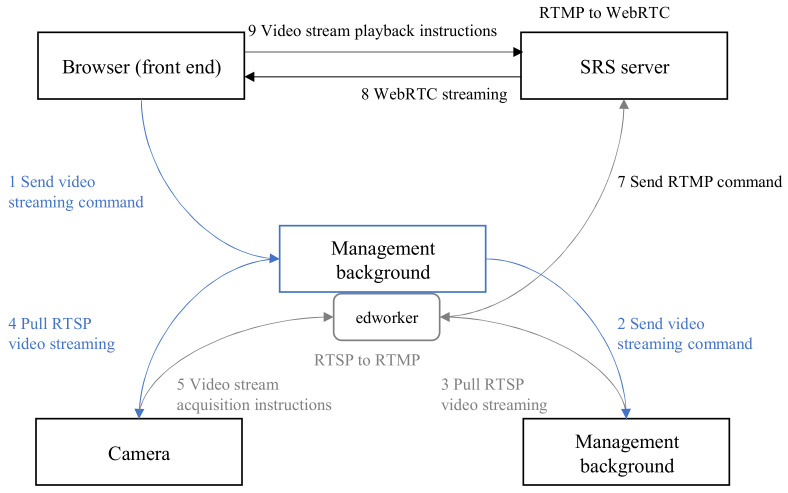
Video streaming of sensor and sensor-based data management strategies.

The application system communicates the parameter data between the cloud and the edge side according to the process shown in Figure 3. After receiving the parameter data from sensors in the entire application system, the edge cloud starts forwarding the parameter data to the local edge node set *N*, and at the same time starts running an artificial intelligence model in the application system to calculate traffic flow parameters in real time. Therefore, the total operation time *T* of the current task is:

(2) $$T=\frac{\sum_{i\in N}\sum_{k\in S}t_{i,k}}{M}$$

Here, *M* represents the total resources of edge nodes.

The terminal sensor must be bound to the edge node in order to use its various functions. Typically, the various attribute information of the terminal sensor needs to be configured on the web interface first, and then the specified terminal sensor and the specified edge node are bound.

In Figure 3, we have adopted the commonly used SRS service forwarding in the industry instead of direct connection between servers and sensors, which can reduce the transmission delay of traffic flow data. As shown in Figure 3, we propose a video streaming method for the edge computing backend system, the cloud-edge collaborative system, and the management backend hardware program edworker.

The browser sends video stream instructions to the edge computing backend and synchronizes them to the cloud-edge collaborative system. The edge computing backend sends RTSP video stream pull instructions to the terminal video sensor based on the agreed instruction set with the cloud-edge collaborative system.

### 4.3. Node Dynamic Scheduling Mechanism and Optimization Strategy Method

PSO is a random searching optimization algorithm that relies on group collaboration, inspired by in-depth research on bird foraging behaviors. Obtaining the optimal solution to the problem, the core idea is to simulate the collective intelligence behaviors exhibited by birds during foraging, and to find the optimal path by simulating the cooperation mechanism between bird flocks.

In the PSO algorithm, each problem to be solved is treated as a “particle” in the searching space, representing candidate solutions with fitness, velocity, and position attributes. Velocity guides the direction and distance of the movement. Seeking the optimal solution, the core of particle swarm optimization algorithm lies in driving the entire population from disorder to order in the searching space through information sharing and collaboration among individuals. The particle update formula is:

(3) $$v_{i,k+1}=\omega \times v_{i,k}+c_{1}\times r_{1}\times \left(l_{i,opt}-x_{i,k}\right)+c_{2}\times r_{2}\times \left(g_{i,opt}-x_{i,k}\right)$$

Here, *i* represents the i-th particle, and *v* and *x* are the particle velocity and position; *k* is the inertia factor, adjusting the global and local iterative optimal strategies, and ω represents the search step size, also known as the inertia coefficient; $c_{1}$ and $c_{2}$ are learning factors that regulate the proportion of influence on flight direction, i.e., the proportion of search direction; $r_{1}$ and $r_{2}$ are random values between 0–1 that follow a uniform distribution; $c_{1}\times r_{1}\times \left(l_{i,opt}-x_{i}\right)$ is the particle’s own dependencies and $c_{2}\times r_{2}\times \left(g_{i,opt}-x_{i}\right)$ is the particle team dependencies.

In this article, the relationship between applications and nodes can be represented using PSO. The loading and unloading of models in nodes is the process of particle swarm optimization searching for optimal solutions. Unlike particle updates, we need to impose constraint conditions on the optimal solution. In addition to meeting the limitations of node resource conditions on applications, it is also necessary to consider the dependency relationship between application data source sensors and application edge artificial intelligence models.

The node has been represented by the set *N*, defined as in Section 4.1. The number of resources of a node includes CPU/GPU frequency, storage space, power consumption, etc. Performance indicators of nodes include data transmission latency, model inference time, and resource utilization.

The application is represented by the set $A=\left\{a_{1},\;a_{2},a_{3},\ldots ,a_{n}\;\right\}$, which relation with task defined as in Section 4.1. There is a dependency relationship between tasks, sensors, and nodes. Different tasks are processed, analyzed, and predicted using different edge artificial intelligence model algorithms, and their data sources depend on sensors. The dependency relationship between tasks and nodes is not only limited by the number of resources of a single node device, but also related to the task attributes, that is, the source of sensor data. Therefore, the application task is represented by a binary $\left(p_{i},R_{i}\right)$. $p_{i}$ represents the performance metric of the *i*-th application, and $R_{i}$ represents the dependency relationship of the *i*-th application.

Adaptive matching constraints: including matching constraints and data privacy. The former refers to the adaptive matching problem between tasks, sensor data, and node models, while the latter refers to whether sensor data are only processed in edge nodes or can be cloud-edge collaborative.

Therefore, the optimal strategy for this paper is:

(1) Introduce a gain coefficient *g* to automatically adjust the weights of $r_{1}$ and $r_{2}$ in the formulation (3) and $g=2\times \frac{r_{1}}{r_{1}+r_{2}}$

(2) Introduce an adaptive inertia factor to balance the search speed and convergence accuracy of PSO algorithm and $\omega =\omega_{max}-\frac{\omega_{max}-\omega_{min}}{t_{max}}$

Here, $\omega_{max}$ represents the maximum search step size, with a value of 0.99. $\omega_{min}$ represents the minimum search step size, with a value of 0.1. $t_{max}$ represents the maximum number of iterations, with a value of 1000.

(3) When introducing adaptive matching constraint functions for loading, unloading, and resource scheduling, it is necessary to consider the correlation matching problem between applications and sensors, which is a strong constraint and higher than other constraint conditions. Adding it as a penalty term to the total cost calculation, the adaptive matching constraint function can be expressed as follows:

(4) $$P_{t}=\max\left(C_{i}\right)+corr(R_{i})$$

Here, $C=\{\;f_{i}^{k}<f_{m},\;c_{i}^{k}<c_{m},\underset{k\in S}{\sum }t_{i,k}<T\}$ represents all performance constraints (including resource and privacy constraints) of the current application, and *corr* represents the covariance correlation coefficient of each dependent component of the application.

The PSO algorithm steps in this paper are as follows:

(1) Initialize, reset all node particle swarm and performance metrics, and calculate adaptive matching constraints.

(2) According to the strategy presented in this article, optimize the application deployment of global and self particles, as well as the current particle execution task.

(3) Update the optimal particle fitness value and global fitness value for the current application.

(4) If the constraint conditions are met, update the gain coefficient and inertia factor; otherwise, update the scheduling strategy or perform unloading operations based on constraint conditions.

(5) Continue the loop until the convergence condition is met; otherwise, jump to (2) and continue.

## 5. Application Cases of AI Technology in Intelligent Transportation Systems

**Figure 4:**
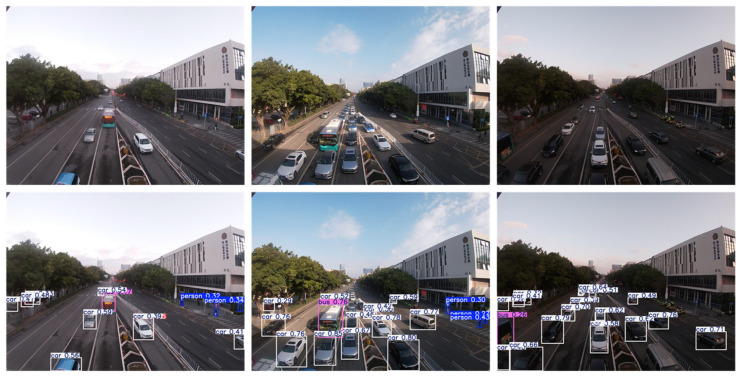
Object detection with edge computing equipment node.

The edge artificial intelligence model can be applied to other fields of intelligent transportation systems. Taking Figure 4 as an example, it shows the experimental results of deploying object detection models (such as YOLO series models) on edge node devices to achieve tasks such as vehicle object recognition and tracking. By using NVIDIA accelerated inference supported on edge smart devices, the computation process of object detection can be optimized in terms of memory, parameters, etc., thereby achieving real-time inference.

As shown in Figure 4, We used UAV to collect traffic data on urban roads in Shenzhen, and used Yolo v8 model to perform real-time detection of dynamic traffic flow on the same road section. The results indicate that traffic flow changes frequently at different time periods (with varying light intensities), but traffic objects can be well detected in edge devices.

In Figure 4, the effect of deploying the object detection model on edge intelligent node is the result of multiple functions working together. Firstly, at the beginning of model establishment, the model network is trained using confidence loss function and classification loss function, which is shown in the following equation:

(5) $${\mathrm{Loss}}_{\mathrm{obj}}=-\frac{1}{N}\overset{N}{\underset{i=1}{\sum }}\left(y_{i}\log\hat{y_{i}}+\left(\left(1-y_{i}\right)\log\left(1-\hat{y_{i}}\right)\right)\right)$$

(6) $${\mathrm{Loss}}_{\mathrm{cls}}=-\frac{1}{N}\overset{n}{\underset{i=1}{\sum }}\overset{C}{\underset{c=1}{\sum }}1_{\left\{c=y_{i}\right\}}\log({\hat{p}}_{\mathrm{ic}})$$

By backpropagation of the loss function, the weights of various parts such as model confidence, classification, and IOU are optimized and adjusted to ultimately retain the model with better training. By optimizing and deploying the model through the model acceleration inference technology carried by edge intelligent devices, real-time tasks such as object detection and tracking can be performed.

The model inference stage is a cyclic process that continuously obtains the confidence level of the object position and category, and generates a score as the best confidence level for the current candidate box. Then, through maximum suppression, candidate boxes with a high probability of being duplicate targets are removed, and finally all recognized objects are retained.

The Volta architecture in edge smart devices has added optimization support for Tensor Cores, with inference computing power far exceeding that of the GTX 1080 Ti on the PC. At the same time, even though the number of CUDA cores and Tensor Cores is significantly lower than that of RTX 3080 Ti, the inference performance can still approach RTX 3080 Ti, demonstrating excellent computational efficiency and low power consumption advantages. Using the CUDA computing power of GTX1080Ti as the standard, compare and quantify the computing power of edge intelligent devices and RTX3080Ti, as shown in Table 2.

We evaluate the effectiveness of object detection based on the recall rate, and the calculation formula is as follows:

(7) $$R=\frac{TP}{TP+FN}\times 100\%$$

Here *R* is the recall rate; *TP* (true positional) refers to the correct retrieval of positive samples as positive samples; and *FN* (False Negative) means that positive samples are erroneously retrieved as negative samples.

In addition, based on the same model and experimental data, quantitative analysis of object detection results under the three architectures showed almost identical detection rates, while edge devices saved 90% to 217% of computing resources.

**Figure 5:**
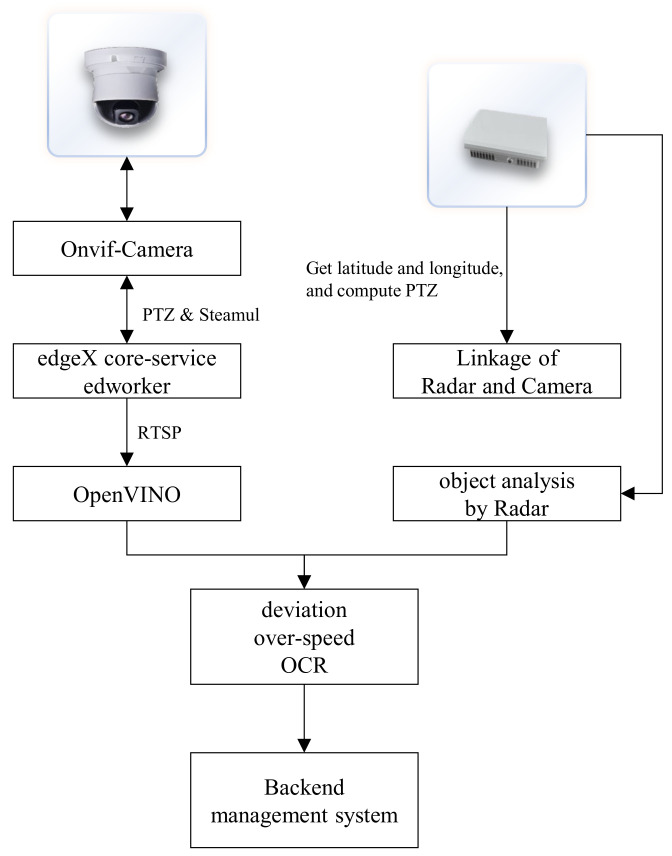
Artificial intelligence model application process in bridge application system.

This paper takes a bridge intelligent transportation system as an example to systematically demonstrate the specific implementation process and system design of AI models in an edge computing platform. This system has open data interfaces and reserved interfaces and fields to ensure system scalability, facilitating the integration with other bridge information systems. By connecting with relevant local maritime bureaus, navigation bureaus, emergency departments, and bridge owners, it provides departments with data such as ship traffic information and bridge ship warning information. Figure 5 mainly introduces the implementation process of the artificial intelligence model in the bridge application system. According to its process, the technical route and implementation process are shown in here.

The Bridge Intelligent Anti-Collision Warning Platform, based on the “end, edge, cloud, intelligence” architecture of edge computing, can be divided into the following four parts:

### 5.1. Sensor Access at the Endpoint

(1) Cameras are accessed through ONVIF and registered in the edgeX 2.0 core services.

(2) Radar network port data are read through customized services.

### 5.2. Core Programs at the Edge

(1) Uses edgeX 2.1 ARM version, deployed on Nvidia’s edge intelligence box.

(2) Composition of edgeX services: core-data, command, metadata, ekuiper.

(3) Develops the edmonitor service, compiles it into a Docker image, and uploads it to the private repository, then is deployed on the edge intelligence box with edgeX services.

### 5.3. Cloud Management Platform

(1) Frontend developed with Vue; backend developed with Java 1.8.0, Golang 1.16, and C/C++ 14, with interactions between frontend and backend via HTTP and WebSocket.

(2) Acquires the internal RTSP stream of cameras through edgeX, uses ffmpeg to convert RTSP addresses to public stream proxy programs, and displays video streams through WebRTC on the page.

### 5.4. Ship Behavior Detection

(1) Uses OpenVINO 2021.4.752 version combined with millimeter-wave radar for ship deviation, over height, and over speed.

(2) Stores abnormal results locally in Redis and reports abnormal information to cloud programs through interface programs.

**Figure 6:**
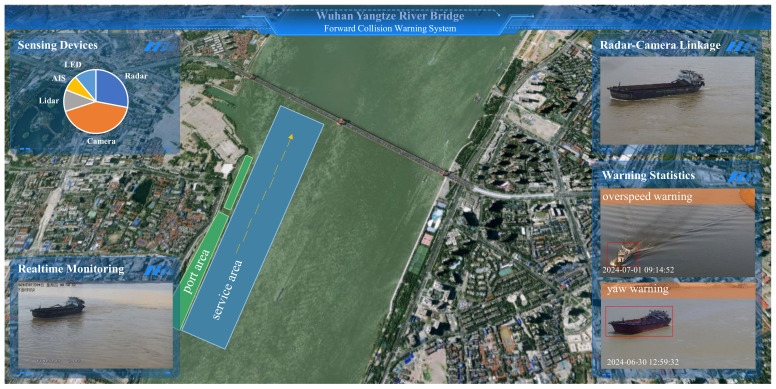
Bridge Intelligent Anti-Collision Warning Platform with sensor-Based data.

Figure 5 illustrates the application of artificial intelligence models in intelligent transportation systems, which utilize sensors to perceive traffic flow data and apply artificial intelligence models to achieve object detection of ships. The active bridge collision warning platform based on AI technology and bridge business systems relies on the edge computing platform and bridge business application system. It has realized the detection of over height, over speed, and deviation warnings for ships. The backend management system uses various communication methods for remote reminders of ship warning messages, bridge and surrounding 3D geographic information, hydrological environment, and meteorological information, as shown in Figure 6.

**Figure 7:**
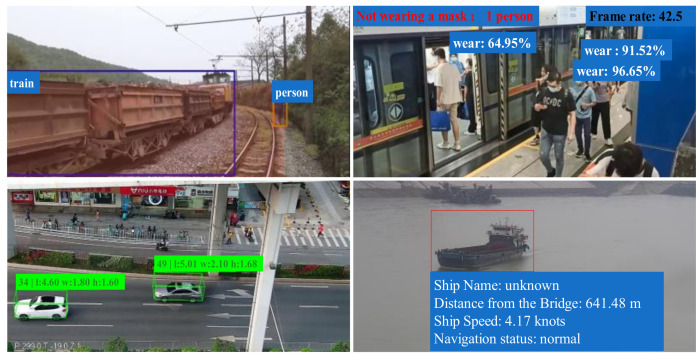
Artificial intelligence model application cases in transportation system.

In addition to realizing bridge business applications, the edge computing AI business application system also integrates AI models for perimeter breach protection, mask recognition and population statistics, vehicle object multimodal recognition, and ship object tracking. The implementation results of other algorithm models of the business system are shown in Figure 7. All the data are experimental data collected by ourselves, including data collected by unmanned aerial vehicle (UAV), mobile phones, and cameras, and the data collection locations in Figure 6 are Shenzhen, Guangzhou, Guangzhou, and Nanning, respectively.

## 6. Summary

This section provides a detailed analysis of the experimental results obtained from the implementation of the proposed automatic AI model loading and unloading strategy in an edge computing environment. The evaluation focuses on key performance metrics, including latency, resource utilization, model efficiency, and data privacy improvements. The results demonstrate the effectiveness of the proposed strategy in optimizing resource utilization and operational efficiency while enhancing data privacy protection.

(1) Latency: Latency is a critical metric in intelligent transportation systems, as real-time decision-making is essential for effective traffic management. The proposed strategy significantly reduces latency by dynamically loading and unloading AI models based on edge device requirements. Experimental results show that the average latency for processing traffic data decreased by 25~35% compared to traditional static model deployment approaches. This improvement is attributed to the efficient allocation of computational resources, deployed lightweight models, and the prioritization of high-frequency, low-latency tasks on edge devices.

(2) Resource Utilization: The proposed strategy optimizes resource utilization by dynamically managing the loading and unloading of AI models based on their usage frequency, resource consumption, and task requirements. The experimental results show that compared with traditional methods, the resource utilization efficiency has been improved by about 60%. In edge devices, we used 16 GB of memory and 8 GB of shared video memory with SOC graphics cards. On the server side, 32 GB of memory and 22 GB of dedicated graphics card were used.

(3) Model Efficiency: The strategy emphasizes the use of lightweight models and model compression techniques to ensure compatibility with edge devices’ hardware architecture and operating systems. The experimental results demonstrate that the proposed approach saves 90% to 217% of computing resources without compromising accuracy.

(4) Data Privacy Improvements: Data privacy is a significant concern in intelligent transportation systems, as sensitive traffic data must be protected from unauthorized access. The proposed strategy enhances data privacy by processing sensitive data locally on edge devices and only transmitting anonymized or aggregated data to the cloud. In our experiment, over 90% of the data was processed and modeled on local edge devices, greatly reducing the security and privacy issues of data exposure.

The experimental results validate the effectiveness of the proposed automatic AI model loading and unloading strategy in optimizing resource utilization, reducing latency, improving model efficiency, and enhancing data privacy in intelligent transportation systems. The strategy’s ability to dynamically manage models based on real-time requirements ensures efficient operation in edge computing environments. Future research will focus on further refining cloud-edge collaborative training methods and exploring additional optimization techniques to enhance the system’s performance and scalability.

## 7. Future Work

The main focus of this paper is studying on automatic loading and unloading strategies for models under the condition of resource collaboration between multiple nodes and tasks. It is more suitable for offline data analysis and model application of edge nodes. It does not consider the collaboration of cloud-edge data and the self-learning mechanism of artificial intelligence models.

The advantage of cloud-edge collaborative training lies in fully leveraging the respective strengths of cloud and edge devices. The cloud possesses powerful computational capabilities and abundant data resources, suitable for large-scale training and optimization of AI model of intelligent transportation. On the other hand, edge devices offer advantages in real-time processing and data privacy protection, enabling preliminary data processing and AI model inference locally. By coordinating the work between the cloud and edge devices, more efficient, accurate, and secure AI applications can be achieved.

In the future, we will explore combining cloud computing and edge computing through cloud-edge collaborative training methods. The cloud will handle the training of large AI models of intelligent transportation, while edge devices will be responsible for IOT data collection, preliminary data processing, and AI model inference. Efficient communication protocols will facilitate data transmission and model updates between the cloud and edge devices, achieving real-time model optimization and iteration.

## Figures and Tables

**Table 1:** Explanation and usage of specialized terms.

Definition	Explanation
node	Edge computing equipment
sensor	Cameras, millimeter wave radar, LiDAR, etc.
Cloud-Edge collaboration	Cloud and edge collaboration, including resource collaboration, application, data, intelligent collaboration, and other tasks
Video streaming	The process of sending real-time video data
OpenVINO	Open visual reasoning and neural network optimization
WebSocket	A communication protocol on a single TCP connection
edworker	Applications in edge nodes
edmonitor	Applications in edge nodes
SRS	Simple Realtime Server
Redis	Remote Dictionary Server
edgeX 2.0	Open-source edge computing IoT software framework project operated by Linux Foundation
OCR	Optical Character Recognition

**Table 2:** Comparison of edge device and PC with computing power and accuracy.

Platform	Architecture	CUDA Cores	Tensor Cores	FP32(TFLOPS)	Computing Power	R
NVIDIA GTX 1080Ti	Pascal	3584	None	11.34	100%	77.8%
ECone EB122NN	Volta	384	48	6–8	10%	77.8%
NVIDIA RTX 3080Ti (all supplied by NVIDIA Corporation, Santa Clara, CA, USA)	Ampere	8704	332	34.1	227%	77.8%

## Data Availability

The data presented in this study are available on request from the corresponding author due to commercial application data.
